# Genome Sequence of Escherichia coli Clone O25:H4 Sequence Type 131, Isolated from a Sudanese Patient with Urinary Tract Infection

**DOI:** 10.1128/MRA.01326-19

**Published:** 2020-03-12

**Authors:** Sofia B. Mohamed, Mohamed M. Hassan, Sumaya Kambal, Abdalla Munir, Nusiba I. Abdalla, Ahmed Hamad, Sara E. Mohammed, Fatima E. Ahmed, Omnia Hamid, Arshad Ismail, Mushal Allam

**Affiliations:** a Bioinformatics and Biostatistics Department, National University Biomedical Research Institute, National University, Khartoum, Sudan; b Sequencing Core Facility, National Institute for Communicable Diseases, National Health Laboratory Service, Johannesburg, South Africa; Loyola University Chicago

## Abstract

We report here the whole-genome sequence of Escherichia coli NUBRI-E, a representative of E. coli clone O25:H4 sequence type 131 with *bla*_CTX-M-15_, which was obtained from a Sudanese patient with a urinary tract infection.

## ANNOUNCEMENT

Escherichia coli is the most common uropathogen associated with urinary tract infections globally, including Sudan (1–3). Uropathogenic E. coli (UPEC) strains harbor virulence factors, which are usually encoded on pathogenicity islands, providing a mechanism for coordinated horizontal transfer of virulence genes (4, 5). Furthermore, the presence of multidrug-resistant UPEC strains harboring several virulence factors is a major risk factor for inpatients (6). Here, we report the draft genome sequence of E. coli clone O25:H4 sequence type 131 (ST131) (NUBRI-E), which was isolated in Sudan.

A urine sample was collected, via the clean-catch method, from a 22-year-old male patient who had been admitted to the Omdurman Teaching Hospital in Khartoum, Sudan, with a urinary tract infection. The specimen was inoculated directly onto MacConkey agar and then incubated overnight at 37°C under aerobic conditions. The colony was identified using Gram staining and biochemical tests, including the oxidase test, catalase test, Kligler’s iron agar test, sulfide indole motility test, citrate agar test, and urea test. The Analytical Profile Index was used to confirm the species (7).

The genomic DNA was extracted using a QIAamp DNA minikit (Qiagen, Germany). Paired-end libraries were prepared using the Nextera DNA Flex library preparation kit, followed by sequencing (2 × 300 bp) on a MiSeq platform (Illumina, Inc., USA). The sequenced reads were quality trimmed using Sickle version 1.33 (https://github.com/najoshi/sickle). Sickle was run using the following parameters: t sanger, q 20, and l 75. *De novo* assembly was performed using SPAdes version 3.11.1 (8). SPAdes was run using the parameter only-error-correction. All resultant contigs were then submitted to GenBank, where gene annotation was implemented using the NCBI Prokaryotic Genome Annotation Pipeline (PGAP) version 4.8 (9). Serotyping (O-antigen and flagellin genes) was performed with SerotypeFinder version 2.0 (10), multilocus sequence typing (MLST) with mlst (https://github.com/tseemann/mlst), and antibiotic resistance gene determination with ResFinder version 3.2 (11).

A total of 1,777,192 sequencing reads were obtained. The assembled genome was composed of 156 contigs (55.6-fold coverage), all of which were longer than 200 bp (the longest contig was 511,930 bp), covering 5,239,013 bp, with a GC content of 50.7% and *N*_50_ value of 182,864 bp. The NUBRI-E genome was found to harbor 5,050 protein-coding genes, 89 RNA genes, and 176 pseudogenes, as predicted by the NCBI PGAP. The O- and H-antigen types of NUBRI-E are O25 and H4, respectively. MLST based on the seven-allele scheme (E. coli scheme 1) indicated ST131. ResFinder was used to identify acquired antimicrobial resistance genes, with default parameter settings. Multiple antimicrobial resistance genes, including aminoglycoside resistance genes [*aadA5* and *ant(3)-Ia*; a β-lactamase gene, *bla*_CTX-M-15_; a macrolide resistance gene, *mph*(A); a multidrug resistance gene, *mdf*(A); a tetracycline resistance gene, *tetA*; a trimethoprim resistance gene, *dfrA17*; and a sulfonamide resistance gene, *sul1*], were detected in the NUBRI-E genome. To our knowledge, this is the first reported whole-genome sequence of an extended-spectrum-β-lactamase-producing E. coli strain isolated in Sudan.

### Data availability.

This whole-genome sequence project has been deposited at DDBJ/ENA/GenBank under accession number SOYU00000000. The version described in this paper is the first version, SOYU01000000. The sequencing reads have been deposited under SRA accession number SRP218296.
